# Implementation of an Interdepartmental Collaborative Medication Review to Reduce Potentially Inappropriate Medication Use in Hospitalized Older Adults: Protocol for a Mixed Methods Study

**DOI:** 10.2196/69626

**Published:** 2025-07-31

**Authors:** Aravinda Kumar, Rajesh Kumar Konduru, Saranya Rajaram, Manikandan Mani, Anusha Natarajan, Jerin Jose Cherian, Bhavani Shankara Bagepally, Anil Jacob Purty, Nayyar Iqbal, Dineshbabu Sekar, Sudharsanan Sundaramurthi, Isabella Topno, Manjunatha Chalawadi Hanumappa

**Affiliations:** 1 Department of Pharmacology Pondicherry Institute of Medical Sciences Puducherry India; 2 Department of Community Medicine Pondicherry Institute of Medical Sciences Puducherry India; 3 Department of Pharmacology Jawaharlal Institute of Post Graduate Medical Education and Research Puducherry India; 4 Clinical Studies and Trials Unit, Division of Development Research Indian Council of Medical Research New Delhi India; 5 Department of Global Public Health Karolinska Institutet Stockholm Sweden; 6 National Institute of Epidemiology Chennai India; 7 Department of General Medicine Pondicherry Institute of Medical Sciences Puducherry India; 8 Department of General Medicine Jawaharlal Institute of Post Graduate Medical Education and Research Puducherry India; 9 Department of General Surgery Jawaharlal Institute of Post Graduate Medical Education and Research Puducherry India

**Keywords:** collaborative medication review, potentially inappropriate medications, medication-related hospital admissions, older adult prescriptions, facilitators, barriers

## Abstract

**Background:**

The inappropriate use of multiple medications known as polypharmacy is a growing concern for older populations with comorbid conditions in India. Polypharmacy can lead to serious adverse health outcomes, increased health care costs, and reduced quality of life. Screening tools such as the Medication Appropriateness Index (MAI) and Screening Tool for Older Persons’ Prescriptions (STOPP) or Screening Tool to Alert to Right Treatment (START) criteria can help identify potentially inappropriate medications, and interventions such as medication review clinics and prescribing audits can help improve the appropriateness. A collaborative medication review (CMR) involving a team approach is important to ensure that patients receive the best possible care.

**Objective:**

The primary objective is to assess the feasibility of implementation of interdepartmental CMR to reduce potentially inappropriate medications in hospitalized older adults. The secondary objectives are to (1) explore the facilitators and barriers in this implementation from the health care providers’ perspective, (2) determine the costs involved in the implementation from a health system perspective, and (3) analyze the efficacy of interdepartmental CMR by using MAI, postdischarge adverse events, and number of medication-related admissions.

**Methods:**

This study consists of 5 phases aimed at improving CMR practices in India. Phase 1 focused on conducting a scoping review of CMR practices. Phase 2 involved creating standard operating procedures to establish a CMR team, delineating roles and responsibilities, and providing training. Phase 3 will evaluate the efficacy of CMR by using standardized tools such as MAI and STOPP/START criteria. Phase 4 assesses the challenges faced in implementing CMR. Finally, phase 5 analyzes the costs related to CMR implementation. This study employs a multicentered mixed methods approach, combining qualitative methods (in-depth interviews and focus group discussions) to explore implementation challenges and quantitative analysis through a quasi-experimental study involving 280 hospitalized older adults. It aims to measure costs and the reduction of potentially inappropriate medications post discharge.

**Results:**

This study received a grant from the Indian Council of Medical Research–Safe and Rational Use of Medicine Task Force in December 2023. All study preparatory approvals were obtained. Phase 1 and phase 2 were completed by December 2024. Phase 3 is scheduled to finish by June 2025. Phases 4 and 5 are planned for completion by August 2025. Final data analysis and manuscript submission are expected by December 2025.

**Conclusions:**

This study can provide insights into the implementation and effectiveness of CMR and help to understand the facilitators and barriers to implementing interdepartmental CMR and the cost incurred in its implementation. Interprofessional teams will collaboratively review and optimize medications for older patients with multimorbidity in India—a strategy expected to enhance care coordination, improve clinical outcomes, and reduce health care costs.

**Trial Registration:**

Clinical Trials Registry – India CTRI/2024/06/069220; https://ctri.nic.in/Clinicaltrials/pmaindet2.php?EncHid=OTgyNDg=&Enc=&userName=

## Introduction

Inappropriate polypharmacy refers to the use of multiple medications in a way that is not consistent with established treatment guidelines or that can increase the risk of adverse drug reactions, drug interactions, or other health problems. High-risk populations such as older adults and individuals with multiple chronic conditions are particularly vulnerable to inappropriate polypharmacy. In India, inappropriate polypharmacy in older adults is a growing concern due to the lack of awareness and regulations surrounding medication use. The prevalence of potentially inappropriate medicine use among the older Indian adult population is 28% [1]. The older adult population in India represents 8.6% (104 million) of the total population [2]. These patients often have multiple comorbidities, leading to the prescription of multiple medications. The consequences of inappropriate polypharmacy in high-risk populations can be serious and can include increased risk of falls, hospitalizations, and other adverse health outcomes. In addition, inappropriate polypharmacy can lead to increased health care costs and reduced quality of life for patients [3].

Many screening tools are available as either implicit (judgment-based) or explicit (criterion-based) tools to identify inappropriate polypharmacy [4]. Implicit tools such as the Medication Appropriateness Index (MAI) and Assessment of Underutilization of Medicine assess the prescribing quality of a physician’s prescription for an older adult. Explicit tools such as Beer’s criteria and the Screening Tool for Older Persons’ Prescriptions (STOPP) or the Screening Tool to Alert to Right Treatment (START) criteria have been developed from literature reviews, expert opinions, and consensus exercises, typically comprising a list of drugs to be avoided or added for older adult populations [5]. However, along with identifying the inappropriateness, choosing the best intervention to improve appropriate polypharmacy is a priority goal.

Improvements in inappropriate polypharmacy can be achieved by a wide range of interventions to address the complexity of the clinical situations and the individuality of the prescribers. In India, interventions to improve appropriate polypharmacy are needed to address specific problems related to multimorbidity, which need to be integrated into existing health care systems and embedded with interprofessional collaboration. Some of the interventions include educational programs for prescribers or consumers, medication review clinics and specific prescribing audits, and prescribing incentive schemes and regulatory interventions [6].

Effective polypharmacy interventions involving drug reviews require the consideration of several factors, including the hospital setting, health care professional, purpose and scope of review, criteria to guide review, tool to base drug review, and, finally, the method (manual of the clinical decision support system) used for drug review. The current literature distinguishes 3 types of drug reviews, namely, prescription reviews (performed without the patient), concordance and complication reviews, and clinical medication reviews (performed in the presence of the patients). However, in India, it is feasible to conduct only a prescription review on the basis of its convenience and feasibility [7].

Furthermore, drug reviews are usually formalized and driven either by implicit or explicit criteria. Owing to their usefulness, explicit or criteria-based screening tools are used most often to help the systematic assessment of medication appropriateness. Varas-Doval et al [8] conducted the ConSIGUE program, which assessed the impact and implementation of a medication review with follow-up for older patients with multiple medications. They suggested that the provision of a medication review with follow-up in collaboration with general medical practitioners and patients contributes to the improvement of older patients’ health status and reduces their problems related to the use of medicines [8]. Medication reviews have been shown to be a cost-effective strategy through a cost-utility analysis [9]. However, policy makers look for other economic evidence other than the cost-utility analysis in the process of considering a change in the health policy and a payment for the service.

Malet-Larrea et al [10] conducted a cluster randomized trial of a medication review with follow-up to ascertain its economic impact from a national health system perspective over 6 months. The cost-benefit ratio revealed that for every €1 (US $1.17) invested in a medication review with follow-up, a benefit of €3.3 (US $3.87) to €6.2 (US $7.28) was obtained. In fact, other studies [10] presented even greater cost-benefit ratios such as those included in a series of systematic reviews (1:34.61, 1:17.0, 1:25.95, and 1:75.84).

In India, physicians follow the theme of professional isolation in the management of patients with multimorbidity. This practice emanates from the interplay among 4 aspects of patient management, namely, disorganization and fragmentation of health care, inadequacy of guidelines and evidence-based medicine for multimorbidity, challenges in delivering patient-centered rather than disease-focused care, and barriers to shared decision-making.

Interdepartmental collaborative reviews may overcome all these barriers, allowing for a more comprehensive review of the patient’s medication needs, considering factors such as the patient’s medical history, current symptoms, and medication side effects. This study will evaluate the challenges in the implementation of an interdepartmental collaborative medication review (CMR) and the cost analysis of a planned CMR to prevent potentially inappropriate medications in the hospitalized older adult population.

## Methods

### Study Settings

**Figure 1:**
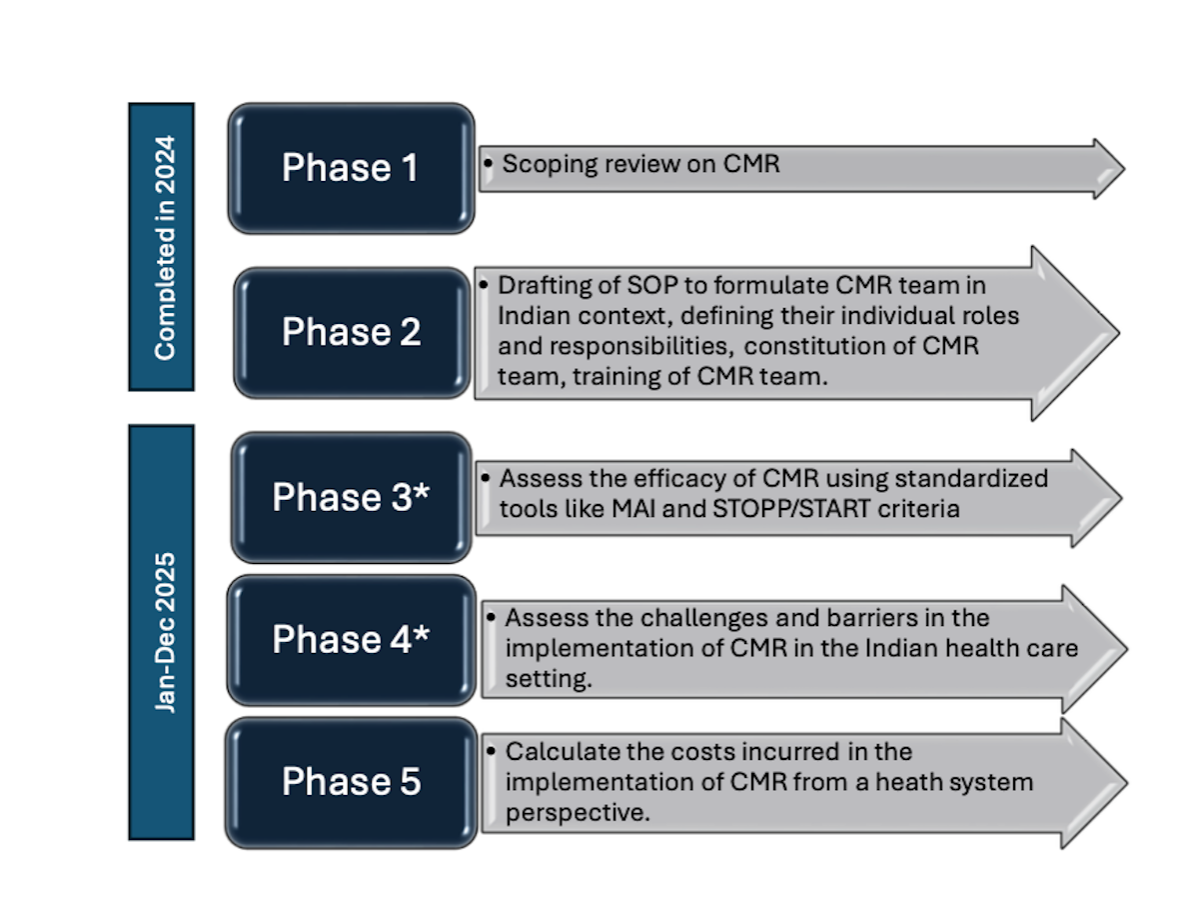
Description of the 5 phases of the study. * denotes phases 3 and 4 will be conducted simultaneously. CMR: collaborative medication review; MAI: Medication Appropriateness Index; SOP: standard operating procedure; START: Screening Tool to Alert to Right Treatment; STOPP: Screening Tool for Older Persons’ Prescriptions.

This study is planned to be conducted over a period of 2 years in 5 phases chronologically (Figure 1). The study will be conducted at the Pondicherry Institute of Medical Sciences and Jawaharlal Institute of Postgraduate Medical Education and Research, Puducherry, India, in collaboration with Indian Council of Medical Research (ICMR)-National Institute of Epidemiology, Chennai, India, and ICMR headquarters, New Delhi. The scoping review (phase 1) on CMR is completed.

### Phase 1: Scoping Review on CMR

We conducted a comprehensive search on databases, including PubMed, Cochrane Library, Clinicaltrials.gov, Global Index Medicus, Lens.org, and World Health Organization (WHO)-International Clinical Trials Registry Platform registries by using the following search terms: medication review, collaborative review, prescriptions, prescription drugs, and aged people. We included only English language studies. Two review authors independently extracted data from each included study via a predesigned data extraction form. We will attempt to contact the trial authors to request incompletely reported data. The following information was extracted: general information (study ID, date of extraction, title, authors, and source of study if not published), study characteristics (study settings, study design, participants, and inclusion/exclusion criteria used in the study), details of interventions, outcomes as described in the types of outcome measures above, and details necessary for the risk of bias assessment. Disagreements between review authors were resolved by discussion or by consultation with a third review author.

### Phase 2: Drafting Standard Operating Procedures to Formulate CMR Teams in the Indian Context

On the basis of the results of the scoping review, the standard operating procedures will be developed by the team of investigators. The scope of the standard operating procedures includes the composition (members) of the CMR team; training of the CMR team; frequency of the CMR team review meetings; details regarding the intervention, which includes the assessment of drug charts and conveyance of information regarding any medication inappropriateness to the treating clinicians as well as follow-up of the participants; and the documentation process. All the developed standard operating procedures were reviewed and approved by the principal investigator and the head of the institution.

### Phase 3: Assess the Efficacy of CMR

The efficacy of CMR is assessed via standardized tools such as MAI, START/STOPP criteria, and AT-HARM10 (Assessment Tool for identifying Hospital Admissions Related to Medications-10 items).

### Phase 4: Assessing the Challenges and Barriers

Phase 4 involves assessing the challenges and barriers in implementing CMR in the Indian health care setting.

### Study Design

#### Mixed Methods Study Design

##### Qualitative Component

In-depth interviews and focus group discussions (FGDs) were conducted to explore the facilitators and barriers to the implementation of interdepartmental CMR to reduce potentially inappropriate medications in hospitalized older patients.

##### Quantitative Component

The study design was quasi-experimental preinterventional and postinterventional with the following components: (1) costs incurred for the implementation of CMR in Indian health care settings and (2) MAI, reduction in the percentage of inappropriate medications, postdischarge adverse events, and medication-related admissions.

#### Study Period and Duration

Each participant’s participation will last from admission until 30 days after discharge from the hospital.

#### Study Participants

Health care providers at the 2 sites will be involved in the study to assess the facilitators and barriers in the implementation of interdepartmental CMR from their perspective. The prescriptions of both male and female older patients (aged 60 years and older) admitted to any medical or surgical ward at both study sites will be assessed to determine whether the participants meet the eligibility criteria after the medical records are reviewed.

### Study Eligibility Criteria

#### Inclusion Criteria

The inclusion criteria were as follows:

1. Male and female patients aged 60 years and older with multiple morbidities admitted to all medical and/or surgical specialty/subspecialty wards at the study sites.
2. Patients visiting the study sites from places (villages/towns) within a 50-km radius (for ease of performing the study and reducing attrition).

#### Exclusion Criteria

The exclusion criteria were as follows:

1. Patients who are discharged against medical advice before the completion of treatment.
2. Patients who are referred to other hospitals for various reasons.
3. Patients or patients whose kin refused to give consent to participate in the study.
4. Readmission and emergency department visits other than drug-related problems.

### Sample Size Estimation and Analysis

The sample size calculation for the assessment of CMR efficacy was based on a previous study [1], which showed that the prevalence of potentially inappropriate medications in the older adult population was 28% in India. We assume that the effect size of the interdepartmental CMR is 10%, power is 80%, and significance level is 5%. The sample size calculation was estimated to be 280 participants. Given that this study will be conducted at 2 different sites, Pondicherry Institute of Medical Sciences and Jawaharlal Institute of Postgraduate Medical Education and Research, 140 participants will be recruited from each site. Universal sampling techniques will be used to enroll participants in this phase of the study. For the qualitative component of the study to assess the implementation challenges of CMR, preferably 2 FGDs will be conducted among CMR team members at each study site. Approximately 10-16 in-depth interviews will be conducted per site until the data saturation point is reached.

### Description of CMR Interventions During the Phase 3 Study

#### Screening Phase

Prescriptions of both male and female older patients (aged 60 years and older) admitted to any medical or surgical ward at both study sites will be assessed to determine whether participants meet the eligibility criteria after the medical records are reviewed.

#### Study Enrollment Phase

Once potential participants are deemed eligible, we will proceed with the enrollment process. The collection of baseline data on participants’ medication profiles, medical history, and other relevant information will be performed. The participants’ demographic details and contact information for follow-up will also be documented. Using tools such as MAI and START/STOPP criteria, we will evaluate the incidence of inappropriate medications after 24 hours of admission at baseline and during hospitalization every third day.

#### Intervention Phase

CMR interventions as per the standard operating procedure will be applied, and all the relevant details mentioned in the CMR activities checklist will be executed. The clinical pharmacists will perform the activities and discuss them with the CMR team, and their suggestions will be presented to the treating physician in written format.

#### Follow-Up Phase

At discharge, the clinical pharmacist will assess the discharge prescription for the MAI score and potentially inappropriate medications. The percentage of acceptance of the suggestions given (by the CMR team during the admission) by the clinicians will also be recorded from the patient records. After discharge, the patients will be telephonically followed up by a trained field investigator for 30 days to inquire about any hospital admissions, visits to the emergency department, drug-related complications, and episodes of dizziness and falls. The AT-HARM10 tool will be used to identify the medication-related hospital admissions after discharge after obtaining the necessary permission from the authors. The detailed description of the study evaluations/measurements/assessments at various time points after CMR intervention is shown in Table 1.

A comparison of MAI scores and the incidence of potentially inappropriate medications before and after CMR intervention at admission and at discharge will predict the efficacy of CMR.

**Table 1:** Description of the study evaluations/measurements/assessments after collaborative medication review intervention.

Event time points			Hospital admission (day 1)		During hospitalization (every third day until discharge)		Hospital discharge		
							Day X^a^		Day 30^b^
**Enrollment**									
	Eligibility screening	✓							
	Informed consent	✓							
**Screening prescriptions**									
	START^c^/STOPP^d^ criteria	✓		✓		✓			
	MAI^e^ score	✓		✓		✓			
**Intervention (CMR^f^)**									
	Medication history	✓		✓		✓			
	Medication reconciliation	✓		✓		✓			
	Medication review	✓		✓		✓			
	Collaborative team meeting	✓		✓		✓			
**Assessments**									
	CMR team time	✓		✓		✓			
	Cost analysis	✓		✓		✓			
	Drug-related problem	✓		✓		✓		✓	
	Hospital admissions							✓	

^a^X refers to the day of discharge of the patient from hospital since admission.

^b^Day 30 refers to 30th day after discharge.

^c^START: Screening Tool to Alert to Right Treatment.

^d^STOPP: Screening Tool for Older Persons’ Prescriptions.

^e^MAI: Medication Appropriateness Index.

^f^CMR: collaborative medication review.

### Process Indicators of Intervention

The following methods have been used to assess the feasibility of implementing interdepartmental CMR: (1) number of interdepartmental CMR meetings, (2) number of CMR consultations per patient, (3) percentage of medication discrepancies/medication-related adverse events identified, prevented, or resolved, and (4) percentage of suggestions given by the CMR team.

### Study End Points

#### Primary End Point

The primary end point was the facilitators and barriers in implementing interdepartmental CMR to reduce potentially inappropriate medications.

#### Secondary End Points

The secondary end points were as follows:

1. Costs involved in the implementation of CMR in Indian health care settings (tertiary health care settings perspective).
2. Change in the number of potentially inappropriate medications from hospital admission to discharge due to CMR.
3. Change in the MAI score from hospital admission to discharge due to CMR.
4. Number of medication-related hospital admissions from the day of discharge to 30 days post discharge.

### Assessment of the Challenges and Barriers in Implementing CMR in the Indian Health Care Setting

FGDs will be conducted among CMR team members, and approximately 10 to 16 in-depth interviews will be conducted with the prescribers whose patients were recruited as the study participants. This would help in understanding prescribers’ and CMR team members’ perceptions and attitudes toward CMR. An FGD via the nominal group technique is a structured variation of a small-group discussion to reach consensus. It gathers information by asking individuals to respond to questions posed by a moderator and then asking participants to prioritize the ideas or suggestions of all group members.

The participants for FGDs will be chosen via a purposive sampling method, that is, team members of CMR. Preferably, 2 FGDs will be conducted among CMR team members at each study site. The total number of participants per FGD will be 4 to 10, and it will be a heterogeneous group depending upon the operational feasibility. Each FGD will be conducted for approximately 40-60 minutes until the data saturation point is reached. The entire proceedings of the FGD will be audio-recorded using an MP3 player after obtaining verbal consent from the participants.

In-depth interviews will be conducted among prescribers, including physicians, intensivists, and senior staff nurses, by using a predesigned interview guide (Multimedia Appendix 1), with topics covering the working process, resources, competences, drug-related problems, intervention effects, and collaboration. Approximately 10-16 in-depth interviews will be conducted per site until the attainment of the data saturation point. Each in-depth interview will be conducted for a minimum of 20-30 minutes. The participants for the in-depth interviews will be selected via a purposive sampling method. The selection of the participants will be considered to ensure representativeness across all professional domains.

### Phase 5: Calculating the Costs Incurred in the Implementation of CMR From a Health System Perspective (Opportunity Costs)

The multitude of health care providers and payment schemes affects the cost of any health service delivered and the estimation methods used, as costs can differ significantly between health centers due to variations in the service type and structure. Accurate cost analysis requires thorough understanding of a health care facility’s functions and financing mechanisms to ensure that all expenses are considered. In this study, a comprehensive case record form will systematically document both direct and indirect costs related to the CMR process. This includes detailed information on the facility characteristics, human resources, infrastructure, equipment, and operational costs, allowing for an extensive evaluation of the economic impact of the CMR process through quantitative and qualitative data collection [11,12].

### Study Variables and Tools

The study variables, namely, patient demographics (name, age, contact information) and medical history, including current and past medical conditions, allergies, and adverse drug reactions, will be collected with the help of a predesigned case record form that includes tools such as the MAI tool, the STOPP/START tool, and the AT-HARM10 tool, which will be used for data analysis. The economic evaluation will be conducted from a health system perspective. Detailed information will be collected on the facility characteristics, human resources, infrastructure, equipment, and operational costs, allowing for an extensive evaluation of the economic impact of the CMR process from a health system perspective. The collected information in the case record forms will also be entered electronically into the Research Electronic Data Capture (REDCap) software (Vanderbilt), improving the data quality and enabling central access to data (since this is a multicentric study).

### Data Management and Statistical Analyses

#### Quantitative Component

The investigators will ensure the completeness, accuracy, and timeliness of data collection. The study sites will enter data in the REDCap software, and case record forms will be scanned in REDCap. The data will be checked for a normal distribution (Kolmogorov-Smirnov test). Student paired 1-sided *t* test will be used to analyze the differences between MAI scores at admission and discharge to determine the effectiveness of CMR, and the chi-square test or Fisher exact test will be used to assess the differences in the frequency distribution. All analyses will be conducted via the SPSS software (version 22.0 for Windows 11; Microsoft), Microsoft Excel 2019, and STATA (version 12; StataCorp LP).

#### Qualitative Component

For the analysis of the qualitative component of the study, the audio-recorded FGDs and in-depth interviews will be transcribed by multiple investigators. The transcripts will be reviewed thoroughly and exported to NVivo software (QSR International). Reflective thematic analysis based on a framework or template approach will be used to analyze the transcripts. Inductive coding of the transcripts will be performed independently by 2 investigators trained in qualitative research, and they will engage in regular analytic discussions to surface assumptions and enhance reflexivity, while field notes will document decisions and researcher positionality. Member checking approach and audit trails will be used to establish credibility and confirmability. A third independent investigator may review the transcribed data to increase the study’s internal validity. Intercoder agreement will be performed to enhance the validity of the study. The codes obtained from the transcripts will be thoroughly reviewed, and the codebook will be generated. The codes will then be merged to form categories, subthemes, and themes and categorized as either facilitators or barriers. Dependability will be addressed through an explicit coding manual and transparent documentation of the analytic steps, while transferability will be supported by providing rich, contextualized descriptions of the participants and selection of study participants.

The statistical analysis to evaluate the costs incurred in implementing CMR in the Indian health care setting from a health system perspective includes direct, indirect, and opportunity costs. Data collected from various facilities will be cleaned and entered into a predesigned database. All costs will be standardized to 2025 prices by using the consumer price index for health care services. Descriptive statistics will summarize continuous variables such as salaries and utility costs by using the measures of central tendency (mean, median) and dispersion (SD, IQR), while categorical variables such as facility type will be reported as frequencies and percentages. Comparative analyses will assess cost differences between public and private sector facilities by using independent-sample 1-sided *t* tests for normally distributed data or Mann-Whitney *U* tests for nonnormally distributed data. To explore the predictors of the cost variations across facilities, we will conduct multivariable linear regression with total CMR costs as the dependent variable and facility characteristics, human resources, and infrastructure costs as the independent variables. Interaction terms such as facility type and operational hours will be included to investigate the moderating effects. All statistical analyses will be performed using STATA (version 18), and *P* values less than .05 will be considered as statistically significant.

### Data Monitoring

Data monitoring will be performed by designated officials appointed by the ICMR headquarters in New Delhi.

### Ethical Considerations

The study was approved by the institutional ethics committee of Pondicherry Institute of Medical Sciences, Puducherry, India (IEC:RC/2023/83 on November 10, 2023) and the institutional ethics committee interventional studies of Jawaharlal Institute of Postgraduate Medical Education and Research, Puducherry, India (JIP/IEC/2024/03/30 on March 20, 2024). This study was registered with the Clinical Trials Registry–India (CTRI/2024/06/069220) on June 19, 2024. All participants will be recruited only after written informed consent is obtained from themselves or their legally authorized representatives. This study conforms to the requirements of the Declaration of Helsinki, 1964; the Indian Good Clinical Practice guidelines; and the ICMR-National Ethical Guidelines for Biomedical and Health Research Involving Human Participants, 2017. Personal information about the screened and enrolled participants will be collected, shared, and maintained to protect confidentiality before, during, and after the trial. Confidentiality of participation will be maintained, and personal identity will not be revealed during data collection, analysis, presentations, and publications. No compensation was provided for participation in the study. Study records will be made available only to authorized persons conducting the study and to the funding agencies without revealing participant identities. All data source documents, including clinical reports and records necessary for the evaluation and reconstruction of the clinical trial, will be stored securely for 5 years, with a focus on ensuring the study participants’ privacy and confidentiality.

## Results

A grant was received from ICMR-Safe and Rational Use of Medicines in December 2023 for this study. This study was scheduled to start from January 2024. The protocol preparation was completed with institutional ethics committee approvals and Clinical Trials Registry–India registrations. The scoping review examining international CMR practices and their adaptability to Indian health care settings was completed adhering to JBI (Joanna Briggs Institute) methodology and PRISMA-ScR (Preferred Reporting Items for Systematic reviews and Meta-Analyses extension for Scoping Reviews) guidelines. The review examined CMR practices in older patients through a comprehensive analysis of 7747 studies published between 2010 and 2024. A systematic search was conducted across PubMed, Cochrane Central Register of Controlled Trials, Clinicaltrials.gov, Global Index Medicus, Lens.org, and WHO-International Clinical Trials Registry Platform by using the following search terms: medication review, collaborative review, prescription drugs, and aged, and only English language studies were included. All retrieved records were imported into Rayyan software for screening. Two reviewers independently screened the titles and abstracts against predefined inclusion criteria: hospitalized patients aged ≥60 years, original research, or protocols evaluating CMR interventions. Full texts of potentially eligible studies were retrieved and assessed independently. Disagreements at any stage were resolved through discussion or a third reviewer adjudication. The review investigated 3 key aspects: CMR team composition, tools for identifying potentially inappropriate medications, and CMR implementation processes in Indian health care settings.

Standard operating procedures for the CMR intervention have been developed through expert panel consultation. The implementation phase will enroll 280 older patients (aged ≥65 years) with polypharmacy (≥5 medications) between January and June 2025. Economic evaluation using time-motion studies and cost analysis will be conducted from July to August 2025, concurrent with semistructured interviews of health care professionals exploring implementation facilitators and barriers. Thematic analysis of qualitative data, cost-effectiveness calculations, and manuscript preparation will be completed by 2025.

Based on the results of this study, modules will be prepared, and workshops will be organized for the prescribers on CMR interventions. The research findings will be presented at national/international conference for large-scale public dissemination. The results of this study will be published in indexed peer-reviewed scientific journals. The full protocol, participant-level dataset, and statistical code will be made publicly available after the completion of the study.

## Discussion

This study will assess the feasibility of implementing CMR in Indian hospitals and identify the key facilitators and barriers to CMR implementation. This study will evaluate the potential for reducing potentially inappropriate medications and medication-related admissions while providing crucial cost-implementation data for the Indian health care context.

Although CMR implementations have been well-studied in Western health care settings, with documented reductions in potentially inappropriate medications and readmissions, this will be among the first studies to systematically evaluate CMR feasibility and implementation costs in Indian health care settings [3,8,13-17]. The protocol’s mixed methods approach will help bridge the knowledge gap between established Western practices and the unique challenges of the Indian health care system. By adopting a health system perspective for economic evaluation, this study will generate data of direct relevance to policy makers and administrators. However, purposive sampling in the qualitative component may introduce selection bias, and the exclusive focus on tertiary care settings may limit the generalizability of the findings to primary or rural health care facilities. Additionally, the relatively short follow‑up period proposed in this protocol will constrain our ability to assess the long‑term sustainability and downstream clinical effects of CMR.

Upon completion of this study, subsequent research will aim to develop and pilot standardized training modules for clinical pharmacists, ensuring consistency in CMR workflows across institutions. Finally, the findings will inform the creation of national guidelines and policies to support the systematic integration of CMR into regular clinical practice throughout India. Policy briefs summarizing key recommendations will be developed for the Ministry of Health and Family Welfare and relevant state health departments.

The findings of this study will redefine medication management practices and pave the way for evidence-based, scalable models of CMR in resource-constrained settings. It will underscore the need for cross-disciplinary collaboration as a fundamental strategy to address multimorbidity in older adult populations, thereby offering valuable insights for policy makers and health care providers aiming to optimize geriatric care.

## Appendix

Multimedia Appendix 1 Interview guide for focus group discussions among the collaborative medication review team and clinicians.

Multimedia Appendix 2 Peer review report by the National Task Force on Safe and Rational Use of Medicine, Indian Council of Medical Research.

## Abbreviations

AT-HARM10: Assessment Tool for identifying Hospital Admissions Related to Medications-10 items
CMR: collaborative medication review
FGD: focus group discussion
ICMR: Indian Council of Medical Research
MAI: Medication Appropriateness Index
PRISMA-ScR: Preferred Reporting Items for Systematic reviews and Meta-Analyses extension for Scoping Reviews
REDCap: Research Data Capture
START: Screening Tool to Alert to Right Treatment
STOPP: Screening Tool for Older Persons’ Prescriptions
WHO: World Health Organization

## References

[1] Bhagavathula AS, Vidyasagar K, Chhabra M, Rashid M, Sharma R, Bandari DK, Fialova D (2021). Prevalence of polypharmacy, hyperpolypharmacy and potentially inappropriate medication use in older adults in India: a systematic review and meta-analysis. Front Pharmacol.

[2] Ageing and health. World Health Organization.

[3] Kiiski A, Airaksinen M, Mäntylä A, Desselle S, Kumpusalo-Vauhkonen A, Järvensivu T, Pohjanoksa-Mäntylä M (2019). An inventory of collaborative medication reviews for older adults - evolution of practices. BMC Geriatr.

[4] Curtin D, Gallagher PF, O'Mahony Denis (2019). Explicit criteria as clinical tools to minimize inappropriate medication use and its consequences. Ther Adv Drug Saf.

[5] Rankin A, Cadogan C, Patterson S, Kerse N, Cardwell C, Bradley M, Ryan Cristin, Hughes Carmel (2018). Interventions to improve the appropriate use of polypharmacy for older people. Cochrane Database Syst Rev.

[6] Sinnott C, Mercer SW, Payne RA, Duerden M, Bradley CP, Byrne M (2015). Improving medication management in multimorbidity: development of the MultimorbiditY COllaborative Medication Review And DEcision Making (MY COMRADE) intervention using the Behaviour Change Wheel. Implement Sci.

[7] Kurczewska-Michalak M, Lewek P, Jankowska-Polańska B, Giardini A, Granata N, Maffoni M, Costa E, Midão L, Kardas P (2021). Polypharmacy management in the older adults: a scoping review of available interventions. Front Pharmacol.

[8] Varas-Doval R, Gastelurrutia MA, Benrimoj SI, García-Cárdenas Victoria, Sáez-Benito Loreto, Martinez-Martínez Fernando (2020). Clinical impact of a pharmacist-led medication review with follow up for aged polypharmacy patients: a cluster randomized controlled trial. Pharmacy Practice.

[9] Jódar-Sánchez Francisco, Malet-Larrea A, Martín José J, García-Mochón Leticia, López Del Amo M Puerto, Martínez-Martínez Fernando, Gastelurrutia-Garralda MA, García-Cárdenas Victoria, Sabater-Hernández Daniel, Sáez-Benito Loreto, Benrimoj SI (2015). Cost-utility analysis of a medication review with follow-up service for older adults with polypharmacy in community pharmacies in Spain: the conSIGUE program. Pharmacoeconomics.

[10] Malet-Larrea A, Goyenechea E, Gastelurrutia MA, Calvo B, García-Cárdenas Victoria, Cabases JM, Noain A, Martínez-Martínez Fernando, Sabater-Hernández Daniel, Benrimoj SI (2017). Cost analysis and cost-benefit analysis of a medication review with follow-up service in aged polypharmacy patients. Eur J Health Econ.

[11] Prinja S, Singh MP, Guinness L, Rajsekar K, Bhargava B (2020). Establishing reference costs for the health benefit packages under universal health coverage in India: cost of health services in India (CHSI) protocol. BMJ Open.

[12] A Handbook of Health System Costing. Ministry of Health and Family Welfare, Department of Health Research, Health Technology Assessment in India.

[13] Houlind MB, Andersen AL, Treldal C, Jørgensen Lillian Mørch, Kannegaard PN, Castillo LS, Christensen LD, Tavenier J, Rasmussen LJH, Ankarfeldt MZ, Andersen O, Petersen J (2020). A collaborative medication review including deprescribing for older patients in an emergency department: a longitudinal feasibility study. J Clin Med.

[14] Anrys P, Strauven G, Boland B, Dalleur O, Declercq A, Degryse J, De Lepeleire J, Henrard S, Lacour V, Simoens S, Speybroeck N, Vanhaecht K, Spinewine A, Foulon V (2016). Collaborative approach to Optimise MEdication use for Older people in Nursing homes (COME-ON): study protocol of a cluster controlled trial. Implement Sci.

[15] Chan WWT, Dahri K, Partovi N, Egan G, Yousefi V (2019). Evaluation of collaborative medication reviews for high-risk older adults. CJHP.

[16] Romskaug R, Skovlund E, Straand J, Molden E, Kersten H, Pitkala KH, Lundqvist C, Wyller TB (2020). Effect of clinical geriatric assessments and collaborative medication reviews by geriatrician and family physician for improving health-related quality of life in home-dwelling older patients receiving polypharmacy: a cluster randomized clinical trial. JAMA Intern Med.

[17] Willeboordse F, Hugtenburg JG, van Dijk L, Bosmans JE, de Vries OJ, Schellevis FG, Elders PJM (2014). Opti-Med: the effectiveness of optimised clinical medication reviews in older people with 'geriatric giants' in general practice; study protocol of a cluster randomised controlled trial. BMC Geriatr.

