# Supplementary material for: Implementation of an Interdepartmental Collaborative Medication Review to Reduce Potentially Inappropriate Medication Use in Hospitalized Older Adults: Protocol for a Mixed Methods Study
Source: JMIR Res Protoc. 2025 Jul 31;14:e69626. doi: 10.2196/69626 (PMC12355137; doi:10.2196/69626)
Supplement: Multimedia Appendix 1 [file resprot_v14i1e69626_app1.docx]

**Interview guide For CMR team members:**

For the record, can you please tell us for how long have you been a part of Collaborative medication Review Team?

Do you have any prior experience in reviewing the medications (before becoming part of CMR team)?

As a clinician, were the prescriptions prescribed by you subjected by review anytime in the past?

- Have you ever experienced a situation in which a medication review (of prescriptions) in hospital was performed?
- Do you think there are benefits of performing medication reviews in hospital? Can you tell me why?
- Do you like being involved in the process of performing medication reviews in hospital? In which way?
- If not, please tell us the factors which make you not like to be involved in the process of reviewing the medications?
- Please elaborate on the pros and cons of having a Collaborative Medications Review (CMR) process in a tertiary care hospital
- How frequently do you think the prescriptions are to be reviewed in order to get the best outcomes in terms of benefit to the patients?
- Please tell us about the challenges you faced while being part of the CMR team!
- In your perception and understanding, what was the level of acceptance of the CMR team by the clinicians in your hospital?
- Which factors can make the implementation of performing medication reviews in hospital a success?
- What do you think is important in the communication process between health care providers and CMR team members involved in performing medication reviews in hospital?

**Interview guide: focus group discussion among the CMR team members**

1. What are the benefits of performing medication reviews in hospital?
2. What are the barriers or disadvantages of performing medication reviews in hospital?

*All the listed barriers and disadvantages are listed by the facilitator in the* *red and green posts and are put on the wall*. Put a sticker on what you think is the most important benefit and the most important barrier.

1. Group discussion: First reactions from the participants. Do people agree with everything? Do people disagree? Are there differences or similarities between team members?
2. *The focus group facilitator addresses m*ultiple barriers as well as disadvantages and would brainstorm on how we could deal with these barriers and focus on solutions through group discussion and exploratory questions.
3. What were the first reactions towards the CMR team members from the clinicians when their prescriptions were reviewed. Do people agree with everything? Do people disagree? Are there differences or similarities between the professional groups?
4. What are the advantages and disadvantages of performing medication reviews in hospital?
5. Were there any unpleasant and untoward incidences experienced by CMR team members due to reviewing of the prescriptions?

**Interview guide: focus group discussion for the CMR team members**

In the third focus group, the research team decided that more focus should be on solutions on how the acceptance and performance of the interdepartmental Collaboration Medication Review Team (CMR) can be improved. Therefore, the interview guide changed slightly:

1. *The green and red posts will display the benefits and barriers/challenges of performing medication reviews in hospital that were mentioned in the in-depth interviews and focus group discussion.* First reactions from the audience. Do you miss anything? Do people agree with everything? Do people disagree? Are there differences or similarities between professional groups?
2. *The focus group facilitator takes the red post its with the barriers and puts them on the wall.* Multiple barriers were mentioned. I would like to brainstorm with you on how we could deal with these barriers and focus on solutions through group discussion and exploratory questions.
3. In your view/expertise, what steps can be taken to improve the acceptance of the “review of the prescriptions” by the treating clinicians?

**Interview Guide for the clinicians:**

1. Are you aware that your prescriptions were reviewed by an expert team in your hospital?
2. Please tell us what was your first reaction when you came to know that your prescriptions were reviewed by expert team in your hospital?
3. Have you ever heard of the prescriptions being reviewed by expert teams in any other hospitals?
4. Have you ever heard of the term, “Interdepartmental Collaborative Medication Review Team (CMR team)”?
5. In your opinion, do you think reviewing the prescriptions given by the clinicians by CMR team is a good clinical practice?
6. If yes, can you please elaborate why do you consider this as a good clinical practice?
7. If No, can you please tell us why do you think it is NOT a good clinical practice?
8. How was the interaction/communication between the CMR team and yourself during the process of CMR review?
9. In your opinion, can you please tell us what are the areas improvement is required as far as the implementation of CMR in the tertiary care hospitals is concerned?
