# Supplementary material for: Implementation of an Interdepartmental Collaborative Medication Review to Reduce Potentially Inappropriate Medication Use in Hospitalized Older Adults: Protocol for a Mixed Methods Study
Source: JMIR Res Protoc. 2025 Jul 31;14:e69626. doi: 10.2196/69626 (PMC12355137; doi:10.2196/69626)
Supplement: Multimedia Appendix 2 [file resprot_v14i1e69626_app2.pdf]

Indian Council of Medical Research

Minutes of 8th Meeting of the members of the National Task Force on Safe and Rational Use of Medicine

Date: 27th July, 2023
Time: 02:00 PM – 5:30 PM

Hybrid Meeting: Venue –ICMR HQ

The following were present during the meeting

Members of the National Task Force

1. Dr. V.P. Kamboj, Ex-Director, CDRI, Lucknow (Chairperson)
2. Dr. Nilima Kshirsagar, Former National Chair Clinical Pharmacology, ICMR Co-chair of National Task Force
3. Dr. C.D. Tripathi, Professor, Pharmacology, GIMS, Noida
4. Dr. Denis Xavier, Professor of Pharmacology, St Johns Medical College, Bangalore
5. Dr. Sujit Chandy, Professor of Pharmacology, CMC, Vellore
6. Prof. S.K. Maulik, Former Emeritus Scientist, Div. of BMS, ICMR
7. Dr. Arun Kumar Sharma, Professor Community Medicine, UCMS, New Delhi
8. Dr Vishnu Rao, Ex-Director, ICMR-NIMS

ICMR secretariat:

9. Dr. Nabendu S Chatterjee, Scientist G & Head, BMS, ICMR
10. Dr. Sudipto Roy, Scientist E (Med), CSTPU, ICMR
11. Mrs. Sowparnika Treasa Sabu, Research Associate-III, BMS, ICMR

Dr. Nabendu S Chatterjee, Head, BMS, welcomed the members. He then requested the chairperson to chair the meeting. Dr. V.P. Kamboj, welcomed all. An overall activity of past works of SRUM task force was mentioned briefly.

Dr. Nilima Kshirsagar explained the background of SRUM project and emphasized that the projects should be specifically reviewed for adequate description, novelty, scalability of the intervention, study design, outcome and impact on indicators specified earlier.

Dr. Sudipto Roy explained to the members regarding the administrative aspects and updates and recap on past activities, upcoming timelines for the recommended proposals and run-of-show of the meeting.

Discussion and comments

The reviewers gave proposal-specific comments for all five project proposals which needed major revisions. The general comments provided by experts were:

- As per the recommendations by TAG in previous meeting, Principal investigators need to include appropriate subject experts with necessary expertise in the project team to strengthen and guide the project implementation.
- The committee unanimously mentioned that intervention is not adequately described in some of the proposals even after many revisions and hand holding by subject experts.

I. Slot#4 Dr. Rajesh Kumar Konduru

Professor & Head, Department of Community Medicine, Pondicherry Institute of Medical Sciences, Puducherry

Title of the proposal: Implementation of inter-departmental Collaborative Medication Review to reduce potentially inappropriate medication use in the hospitalized elderly patients: a mixed method study

Proposal specific comments:

- The committee unanimously suggested that the topic is of huge importance as this idea of Collaborative Medication Review is already practiced in other countries and for oncology in India.
- The PI included senior level faculty from different broad specialties as co-investigators to help in carrying out this study and also included another study site as JIPMER, Puducherry which was not included in the previous proposal.

- In the previous proposal outcomes of the study were not properly described. So in the revised proposal, indicators like Reduction in the percentage of potentially inappropriate medications, Number of medication related admissions and Mediation appropriateness index were included as part of study outcomes.
- The experts suggest that many studies done in western counterpart have demonstrated the positive impact of multidisciplinary healthcare team in various diseases. Not a single study is done so far in Indian setup to study the impact of multidisciplinary healthcare teams comprising of physicians from different departments, pharmacist and nurse. This shows the relevance of the topic in India.
- Since the feasibility of the study is questionable as some of the prescribers won't allow other team to review their treatment pattern and prescriptions, the experts suggested to do a situational analysis first for one year and ICMR can fund for that initially. Later if the study results show a positive impact in the health care sector, further fund can be issued for the project.
- The experts suggested that giving incentives to the prescribers is not ethical which need to be modified in the proposal.
- The experts suggested that fund for Manpower and contingency can be given to the PI for conducting the study and to strengthen the study.

Final recommendation: Recommended subject to review after Situational analysis study for about 12 months to find out the magnitude and determinants of the issue. Conditional to findings of this study, decision can be taken to recommend the intervention phase for next 24 months. Budget recommended for 36 months, but funds for intervention phase will be released after review of situation analysis study.
